# Broad antagonism of coronaviruses nsp5 to evade the host antiviral responses by cleaving POLDIP3

**DOI:** 10.1371/journal.ppat.1011702

**Published:** 2023-10-06

**Authors:** Yang Wu, Mingwei Li, Jin Tian, Haoxin Yan, Yudi Pan, Hongyan Shi, Da Shi, Jianfei Chen, Longjun Guo, Li Feng

**Affiliations:** State Key Laboratory for Animal Disease Control and Prevention, Harbin Veterinary Research Institute, Chinese Academy of Agricultural Sciences, Harbin, China; The Ohio State University, UNITED STATES

## Abstract

Coronaviruses (CoVs) are a family of the largest RNA viruses that typically cause respiratory, enteric, and hepatic diseases in animals and humans, imposing great threats to the public safety and animal health. Porcine deltacoronavirus (PDCoV), a newly emerging enteropathogenic coronavirus, causes severe diarrhea in suckling piglets all over the world and poses potential risks of cross-species transmission. Here, we use PDCoV as a model of CoVs to illustrate the reciprocal regulation between CoVs infection and host antiviral responses. In this study, downregulation of DNA polymerase delta interacting protein 3 (POLDIP3) was confirmed in PDCoV infected IPEC-J2 cells by isobaric tags for relative and absolute quantification (iTRAQ) and Western blotting analysis. Overexpression of POLDIP3 inhibits PDCoV infection, whereas POLDIP3 knockout (POLDIP3^-/-^) by CRISPR-Cas9 editing significantly promotes PDCoV infection, indicating POLDIP3 as a novel antiviral regulator against PDCoV infection. Surprisingly, an antagonistic strategy was revealed that PDCoV encoded nonstructural protein 5 (nsp5) was responsible for POLDIP3 reduction via its 3C-like protease cleavage of POLDIP3 at the glutamine acid 176 (Q176), facilitating PDCoV infection due to the loss of antiviral effects of the cleaved fragments. Consistent with the obtained data in IPEC-J2 cell model *in vitro*, POLDIP3 reduction by cleavage was also corroborated in PDCoV infected-SPF piglets *in vivo*. Collectively, we unveiled a new antagonistic strategy evolved by PDCoV to counteract antiviral innate immunity by nsp5-mediated POLDIP3 cleavage, eventually ensuring productive virus replication. Importantly, we further demonstrated that nsp5s from PEDV and TGEV harbor the conserved function to cleave porcine POLDIP3 at the Q176 to despair POLDIP3-mediated antiviral effects. In addition, nsp5 from SARS-CoV-2 also cleaves human POLDIP3. Therefore, we speculate that coronaviruses employ similar POLDIP3 cleavage mechanisms mediated by nsp5 to antagonize the host antiviral responses to sustain efficient virus infection.

## Author summary

Coronavirus encodes the conserved 3C-like protease nsp5, which has been reported to antagonize interferon (IFN) responses by cleaving a variety of substrates critical for interferon signaling pathways. In this study, POLDIP3 was screened with apparent downregulation post PDCoV infection and identified as a novel antiviral regulator against PDCoV infection. Notably, PDCoV employs its nsp5 to directly cleave POLDIP3 in favor of its replication. We revealed that nsp5 proteins from other coronaviruses, such as porcine epidemic diarrhea virus (PEDV), transmissible gastroenteritis virus (TGEV) and severe acute respiratory syndrome coronavirus 2 (SARS-CoV-2), also had the protease activity to cleave porcine and human POLDIP3. Thus, the findings will deepen our better understanding of pathogenic mechanisms of coronavirus infections and provide nsp5 as an appealing target for the therapeutic design against coronavirus infections.

## Introduction

Coronaviruses (CoVs) are enveloped positive single-strand RNA viruses that belong to the family *Coronaviridae*. According to serological and genotypic characterizations, CoVs are divided into four genera, including *Alphacoronavirus* (*α*-CoV), *Betacoronavirus* (*β*-CoV), *Gammacoronavirus* (*γ*-CoV), and *Deltacoronavirus* (*δ*-CoV) [1]. The 5’-terminal two-thirds of the CoV genome include two open reading frames (ORF1a and ORF1b) that encode two viral replicase polyproteins (ppla and pplab). The two polyproteins are hydrolysed into 10–16 nonstructural proteins (nsps) by papain-like protease (PLpro of nsp3) and 3C-like protease (nsp5 or 3CLpro) and form mature functional nsps through posttranslational modification. The nsp3 is responsible for the proteolytic cleavage of nsp 1–4 and the protein nsp5 is responsible for the processing of other cleavage sites that results in nsp 5–16 [2]. As a member of the *Deltacoronavirus* genus, porcine deltacoronavirus (PDCoV) was first reported in Hong Kong in 2012 and its etiological features were first characterized in the United States in 2014 [3–5]. Similar to other swine enteric CoVs, including transmissible gastroenteritis virus (TGEV) and porcine epidemic diarrhea virus (PEDV), PDCoV has caused serious clinical symptoms characterized by frequent occurrences of diarrhea, vomiting, and dehydration in piglets [4, 6–10]. Clinically, co-infections of PDCoV with PEDV or TGEV are commonly reported in the pig farms, which has caused significant economic losses to the global swine industry. In addition to the economic issues, potential risks of cross-species transmission and public health security have also been posed relative to PDCoV infection [11].

Innate immunity plays a crucial role in host defense against invading pathogens [12,13]. Upon infection, the conserved microbial components, called pathogen-associated molecular patterns (PAMPs), are sensed by cellular pattern recognition receptors (PRRs). During virus replication, viral RNA, as the major type of PAMP, is recognized by Toll-like receptor 3 (TLR3) or retinoic acid-inducible gene-I (RIG-I)-like receptors (RLRs) (e.g., RIG-I and MDA5) and then recruited to the mitochondrial membrane-located adaptor protein MAVS (also called VISA, IPS-1, and Cardif) to form activated polymers. Subsequently, the activated polymers recruit TANK-binding kinase 1 (TBK1) and the inhibitor of nuclear factor kappa B (IκB) kinase (IKK) complex to phosphorylate interferon regulatory factor 3 (IRF3) and IκBα, respectively, leading to the activation of IRF3 and NF-κB accompanied by induction of type I interferon (IFN-I or IFN-α/β) [14–16]. IFN-I is a potent cytokine of critical importance in controlling viral infections and priming adaptive immune responses [17,18]. Following production, IFN-I initiates a positive feed-back loop by binding to their cognate receptors on the cell surface in an autocrine and paracrine manner [19,20] and activates Janus kinase 1 (JAK1) and tyrosine kinase 2 (Tyk2) which phosphorylate the signal transducers and activators of transcription (STAT) 1 and STAT2. Activated STAT1 and STAT2 and interferon regulatory factor 9 (IRF9) form a transcription factor complex termed IFN-stimulated gene factor 3 (ISGF3). Then, ISGF3 is translocated into the nucleus and binds to the IFN-stimulated response elements (ISRE) to induce the expression of IFN-stimulated genes (ISGs), which establish an antiviral state to restrict the infections of invading pathogens [20–24].

DNA polymerase delta interacting protein 3 (POLDIP3), also known as S6 kinase 1 (S6K1) Aly/REF-like target (SKAR), was identified by Hernandes et al. in 2003 [25]. POLDIP3 was a novel protein interacting with the p50 small subunit of human DNA polymerase δ (pol δ) [26]. POLDIP3 is constituted of two independent domains: the APIM domain (residues 53–125) and an RNA recognition motif (RRM) domain (residues 277–357). Accumulating evidence has shown that POLDIP3 is involved in regulating signal transduction pathways in neurodevelopment, neuropsychiatric diseases, cardiovascular diseases, tumors, and other diseases [27–29]. Previous research has identified POLDIP3 as an mTOR/S6K1 effector recruiting S6K1 to cap-binding complex (CBC) mRNA and enhancing translation efficiency [30]. In addition, Kroczynska et al. have demonstrated that POLDIP3 is engaged in critical and essential roles in regulating mRNA translation of IFN-sensitive genes [31], implying a requirement of POLDIP3 for the generation of the antineoplastic effects by IFN-α. However, the regulatory effects of POLDIP3 in virus infection remains unclear.

The present study observed POLDIP3 with obvious downregulation in PDCoV-infected IPEC-J2 cells by isobaric tags for relative and absolute quantification (iTRAQ) and Western blotting analysis. Overexpression of POLDIP3 suppressed PDCoV infection in IPEC-J2 cells, and POLDIP3 knockout enhanced PDCoV infection, indicating POLDIP3 as an innate antiviral molecule. Surprisingly, an antagonistic strategy was revealed that PDCoV encoded nonstructural protein 5 (nsp5) can reduce the POLDIP3 expression via its cleavage at the glutamine acid 176 (Q176), leading to enhanced PDCoV infection by disrupting the antiviral activity of POLDIP3. Notably, consistent with the obtained data in the IPEC-J2 cells model *in vitro*, reductions in POLDIP3 by cleavage were confirmed in intestinal tissues from PDCoV-infected SPF piglets *in vivo*. We found that nsp5 from other coronaviruses, such as PEDV, TGEV, and SARS-CoV-2, also had the protease activity to cleave human and porcine POLDIP3. Collectively, we speculate that coronaviruses employ similar POLDIP3 cleavage mechanisms mediated by nsp5 to antagonize the host antiviral responses to sustain efficient virus infection, which deepened our better understanding of the mechanisms of coronaviruses infection.

## Results

### POLDIP3 is downregulated upon PDCoV infection

**Fig 1:**
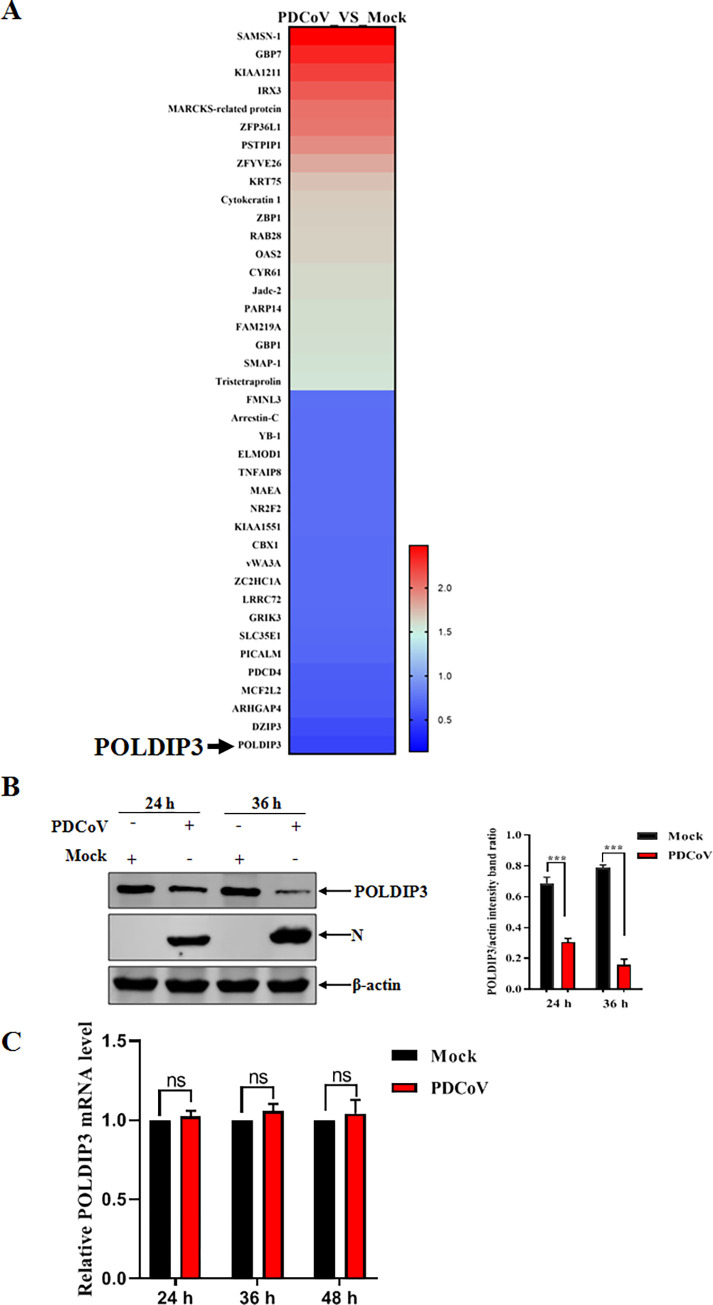
POLDIP3 is downregulated upon PDCoV infection. (A) The iTRAQ analysis of host molecules from host defense upon PDCoV. IPEC-J2 cells were infected with PDCoV (NH strain) at an MOI of 1 or mock-infected with maintenance medium for 36 h, followed by sample collection and iTRAQ analysis. (B) PDCoV infection induced downregulation of endogenous POLDIP3 in IPEC-J2 cells. Target cells were infected or mock-infected with PDCoV (NH strain) at an MOI of 1 and then lysed for detection of endogenous POLDIP3 at indicated timepoints by Western blotting analysis. (C) The regulation of POLDIP3 transcription by PDCoV infection. IPEC-J2 cells were infected with PDCoV as described in panel B and then harvested for detection of POLDIP3 mRNA by RT-qPCR. The results represent three independent experiments (the means ± SD). *, *P*<0.05, **, *P*<0.01, ***, *P*<0.001. The *P* value was calculated using Student’s t-tests.

It is well known that porcine intestinal epithelial cells are the target cells for PDCoV infection *in vivo*. Thus, the IPEC-J2 cell line originating from the porcine intestinal epithelial cell, is an essential model to understand the pathogenic mechanisms of PDCoV infection *in vitro*. To systematically investigate the potential innate immune response to PDCoV infection, host regulators involved in PDCoV infection in IPEC-J2 cells were screened out by iTRAQ assay. Among the regulated host factors, expression of endogenous POLDIP3 protein was significantly downregulated upon PDCoV infection (Fig 1A). To further confirm the results from iTRAQ assay, IPEC-J2 cells were infected with PDCoV or mock-infected as control, followed by the detection of POLDIP3 abundance by Western blotting. As shown in Fig 1B, POLDIP3 abundance was reduced post-PDCoV infection compared to the mock control. Moreover, the POLDIP3 reduction was also observed in attenuated PDCoV-infected IPEC-J2 cells (S1 Fig). Subsequently, we asked whether the POLDIP3 reduction resulted from decreased POLDIP3 transcription induced by PDCoV infection. No significant differences in POLDIP3 mRNA transcription were observed between PDCoV and mock-infected cells at different timepoints (Fig 1C), determined by quantitative reverse transcription-PCR (RT-qPCR). These results suggested that PDCoV infection can downregulate the POLDIP3 expression in IPEC-J2 cells, independent of transcription regulation.

### Ectopic expression of POLDIP3 inhibits PDCoV infection

**Fig 2:**
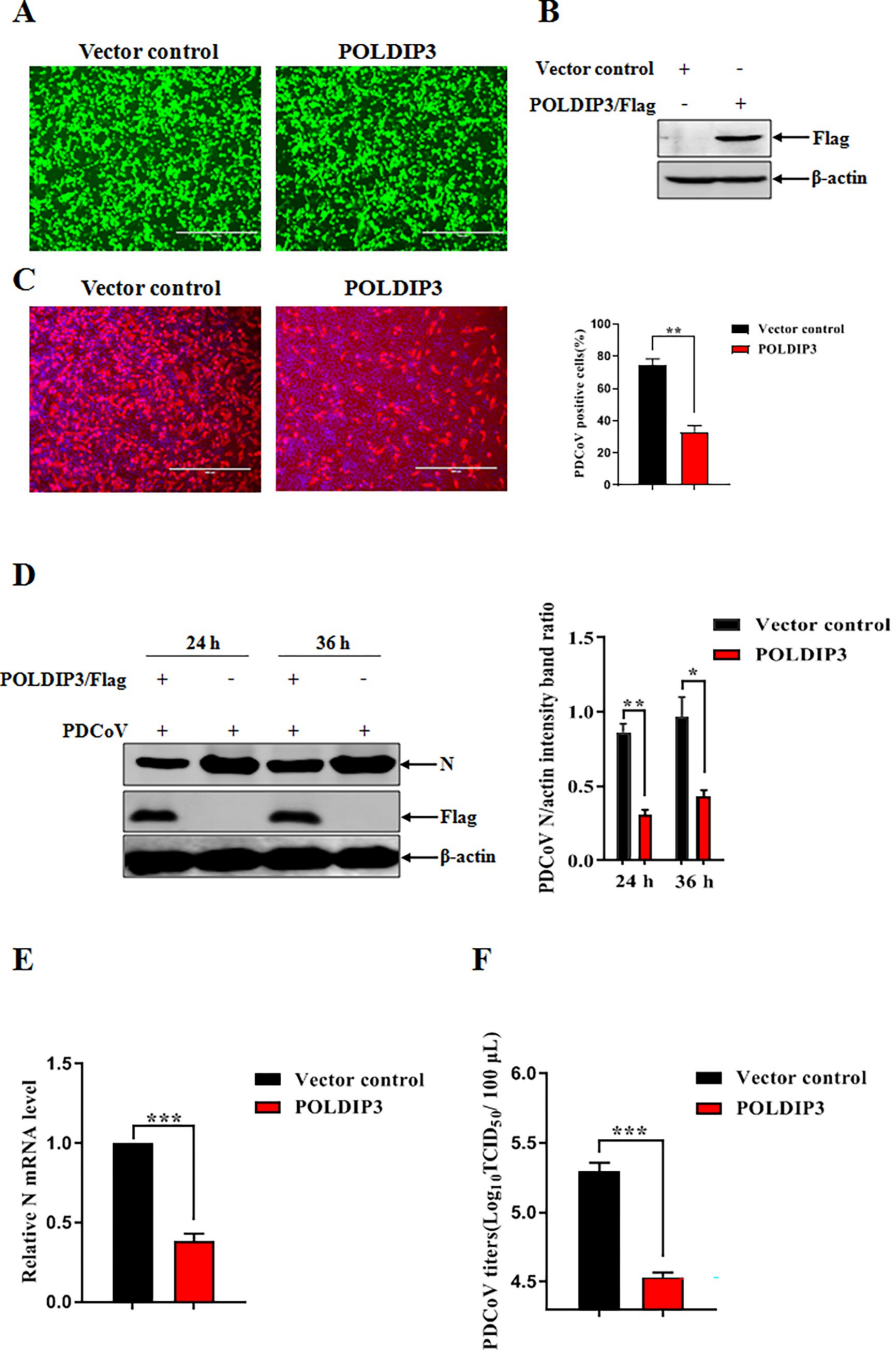
Overexpression of POLDIP3 inhibits PDCoV replication in IPEC-J2 cells. (A) IPEC-J2 cells were transduced with a bicistronic lentivirus vector expressing ZsGreen alone (vector control) or POLDIP3 plus ZsGreen as described in Materials and Methods. (B) Overexpression of POLDIP3 in IPEC-J2 cells. Detergent lysates from cells transduced with POLDIP3 or the vector control were subjected to immunoblotting with the indicated antibodies. (C) Overexpression of POLDIP3 suppressed PDCoV replication by IFA. IPEC-J2 cells were transduced with POLDIP3 or vector control as described above and then infected with PDCoV at an MOI of 1 for 24 h. The percentage of PDCoV-infected cells was determined by immunofluorescence staining. (D) Expression of PDCoV N protein was inhibited by POLDIP3 overexpression. IPEC-J2 cells were transduced with POLDIP3 or vector control as described above and then infected with PDCoV at an MOI of 1 for 24 h, followed by collections of cell samples at 24 h and 36 h post PDCoV infection. PDCoV replication was evaluated by detecting PDCoV N proteins by Western blotting. (E) Ectopic expression of POLDIP3 disrupted PDCoV replication by RT-qPCR. POLDIP3 proteins were overexpressed for 24 h, and then were inoculated with PDCoV (MOI = 1). The level of PDCoV N mRNA was determined at 24 h post PDCoV infection by RT-qPCR. (F) POLDIP3 overexpression decreased PDCoV infection by TCID_50_ assay. Cells were subjected to PDCoV infection for 24 h following POLDIP3 overexpression for 24 h. The virus yield was measured by TCID_50_. The results represent three independent experiments (the means ± SD). *, *P*<0.05, **, *P*<0.01, ***, *P*<0.001. The *P* value was calculated using Student’s t-tests.

Previous studies have showed that POLDIP3 plays an important role in the generation IFN responses [31]. To characterize the involvement of POLDIP3 in the infectivity of PDCoV infection, we investigated the relationship between POLDIP3 expression and virus susceptibility in the IPEC-J2 cell line. IPEC-J2 cells were transduced with a bicistronic lentiviral vector expressing porcine POLDIP3 with a C-terminal Flag tag and ZsGreen (green fluorescent protein), expressed proportionally. The lentivirus encoding ZsGreen alone was included as a vector control. More than 90% of cells were green fluorescence positive, indicating that the lentiviruses were successfully transduced into IPEC-J2 cells (Fig 2A). As shown in Fig 2B, the overexpressed POLDIP3 was confirmed by Western blotting in IPEC-J2 cells. PDCoV infectivity was inhibited in POLDIP3-overexpressing IPEC-J2 cells by immunofluorescence assay (IFA) and Western blotting, respectively, compared to that in vector control-treated parental cells (Fig 2C and 2D). Consistent with the inhibitory effects of viral protein expression, the mRNA levels of PDCoV nucleocapsid (N) were decreased in the treated IPEC-J2 cells with POLDIP3 overexpression by RT-qPCR (Fig 2E). Furthermore, a significant reduction in the titer of progeny virus was confirmed in POLDIP3-overexpressing IPEC-J2 cells relative to the mock cells by the 50% tissue culture infective dose (TCID_50_) (Fig 2F). The data demonstrated that the ectopically expressed POLDIP3 could significantly inhibit PDCoV replication in IPEC-J2 cells.

### Generation of IPEC-J2 knockout cell line lacking POLDIP3

**Fig 3:**
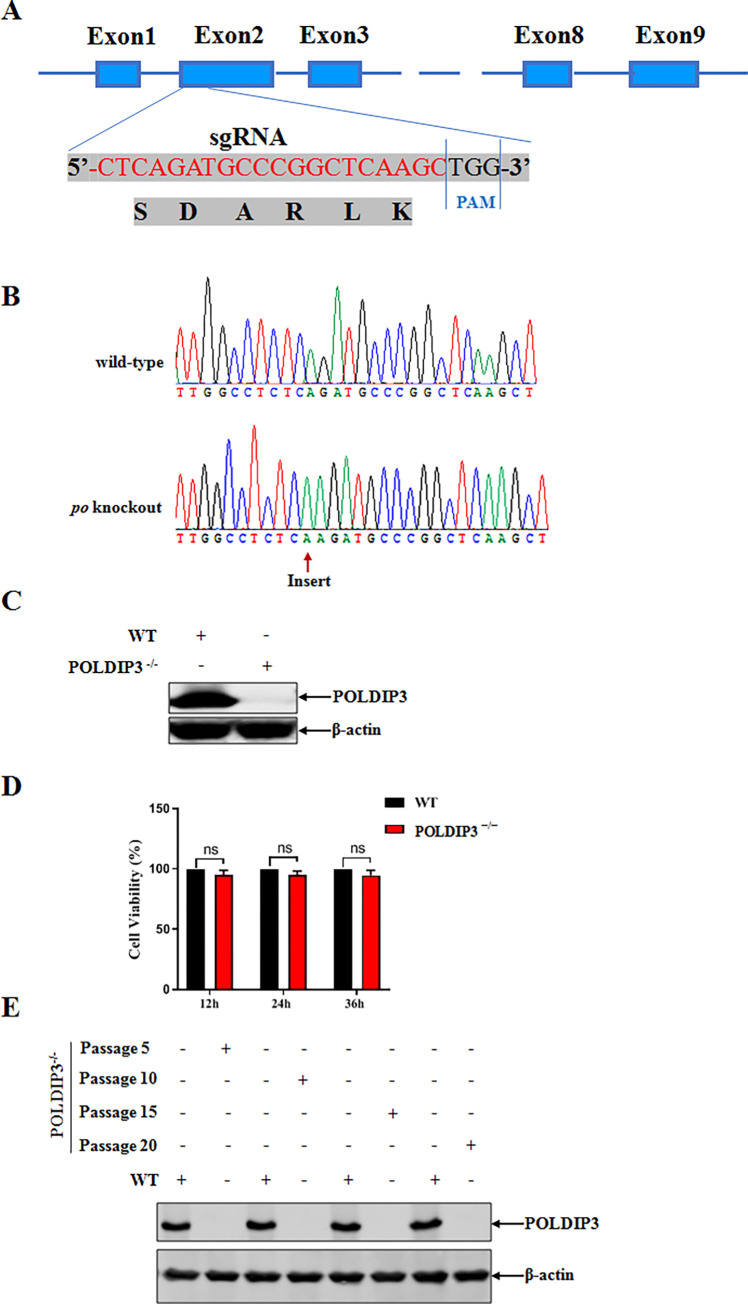
Establishment of POLDIP3 knockout IPEC-J2 cell line. (A) Schematic of the POLDIP3 knockout strategy. The Cas9/sgRNA target sites are indicated in red. (B and C) Confirmation of POLDIP3 knockout in IPEC-J2 cell line using DNA sequencing and Western blotting. (D) Determination of cell viability using CCK-8 detection. (E) Identification of the stability of POLDIP3 knockout cell line by Western blotting.

To study the role of POLDIP3 in PDCoV infection more directly, we generated a single clonal POLDIP3 knockout (KO) IPEC-J2 cell line (POLDIP3^-/-^) using the CRISPR-Cas9 technique. Briefly, the target small guide RNAs (sgRNA) located at the exon 2 of the POLDIP3 gene was designed using the CRISPR Design website (http://crispr.mit.edu) (Fig 3A). The knockout effect of POLDIP3 was determined by sequencing and Western blotting analysis. Three POLDIP3 KO clones were confirmed with a shift of opening reading frame within the POLDIP3 encoding gene by sequencing analysis (Fig 3B), leading to the disruption of POLDIP3 translation as detected by Western blotting (Fig 3C). Meanwhile, knockout of POLDIP3 had no significant effect on the cell viability when compared to the wild-type (WT) cells (Fig 3D). In addition, the POLDIP3 KO cell line was confirmed genetically stable when determined at different passages intervals by Western blotting (Fig 3E).

### POLDIP3 KO cells promote PDCoV infection

**Fig 4:**
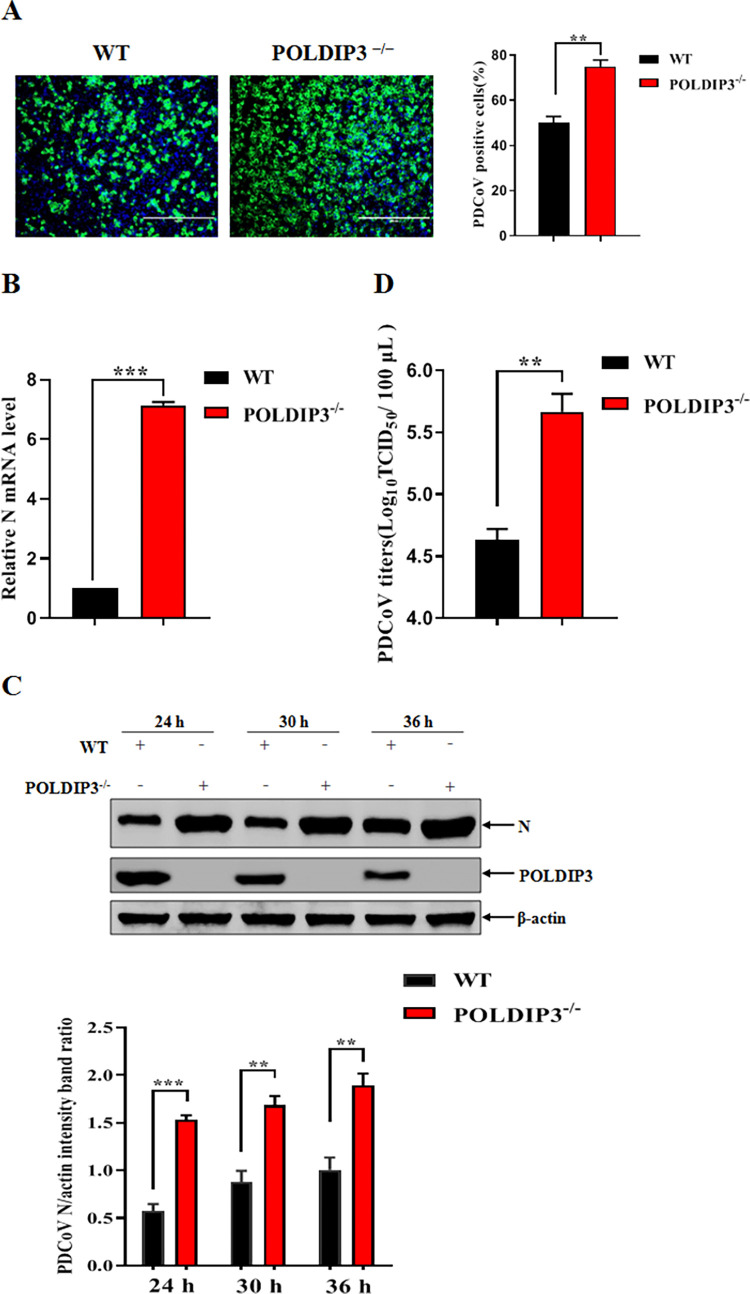
POLDIP3^-/-^ cells promote PDCoV infection. (A) POLDIP3 deletion enhances PDCoV replication by IFA. WT and POLDIP3^-/-^ cells were infected with PDCoV at an MOI of 1. After 24 h post-infection, cells were fixed and stained with antibodies against the PDCoV N protein. The percentage of PDCoV-infected cells was determined by IFA. (B) Knockout of POLDIP3 increases PDCoV replication by RT-qPCR. WT and POLDIP3^-/-^ cells were infected with PDCoV at an MOI of 1 for 24 h, and the levels of PDCoV N mRNA were determined by RT-qPCR. (C) Deletion of POLDIP3 promotes PDCoV replication by Western blotting. WT and POLDIP3^-/-^ cells were infected with PDCoV at an MOI of 1 and the levels of PDCoV N expression were determined at indicated timepoints by Western blotting. (D) POLDIP3 deletion facilitates PDCoV infection by TCID_50_. WT and POLDIP3^-/-^ cells were infected with PDCoV at an MOI of 1 for 24 h, followed by quantification of virus yield by TCID_50_. The results represent three independent experiments (the means ± SD). *, *P*<0.05, **, *P*<0.01, ***, *P*<0.001. The *P* value was calculated using Student’s t-tests.

To assess how POLDIP3 deletion responds to PDCoV infection, POLDIP3^-/-^ or WT cells were infected with PDCoV to evaluate the involvement of POLDIP3 in PDCoV infection. PDCoV replication was significantly increased in the POLDIP3^-/-^ cells compared with that in the WT cells according to IFA (Fig 4A). We then detected the mRNA levels of PDCoV N via RT-qPCR, as shown in Fig 4B, the levels of viral N mRNA were increased in POLDIP3^-/-^ cells compared to WT cells. Consistent with the transcriptional regulation by POLDIP3, the elevated PDCoV N expression was confirmed in POLDIP3 KO cells relative to the WT cells (Fig 4C). In addition, the differences in the yield of progeny virus by POLDIP3 deletion were examined by measuring TCID_50_. The data showed that PDCoV replication was significantly increased (~1 log10) in POLDIP3^-/-^ cells compared to WT cells (Fig 4D), indicating that PDCoV replication was negatively regulated by POLDIP3 expression.

### Complementation expression of POLDIP3 in POLDIP3 KO cells disrupts PDCoV replication

**Fig 5:**
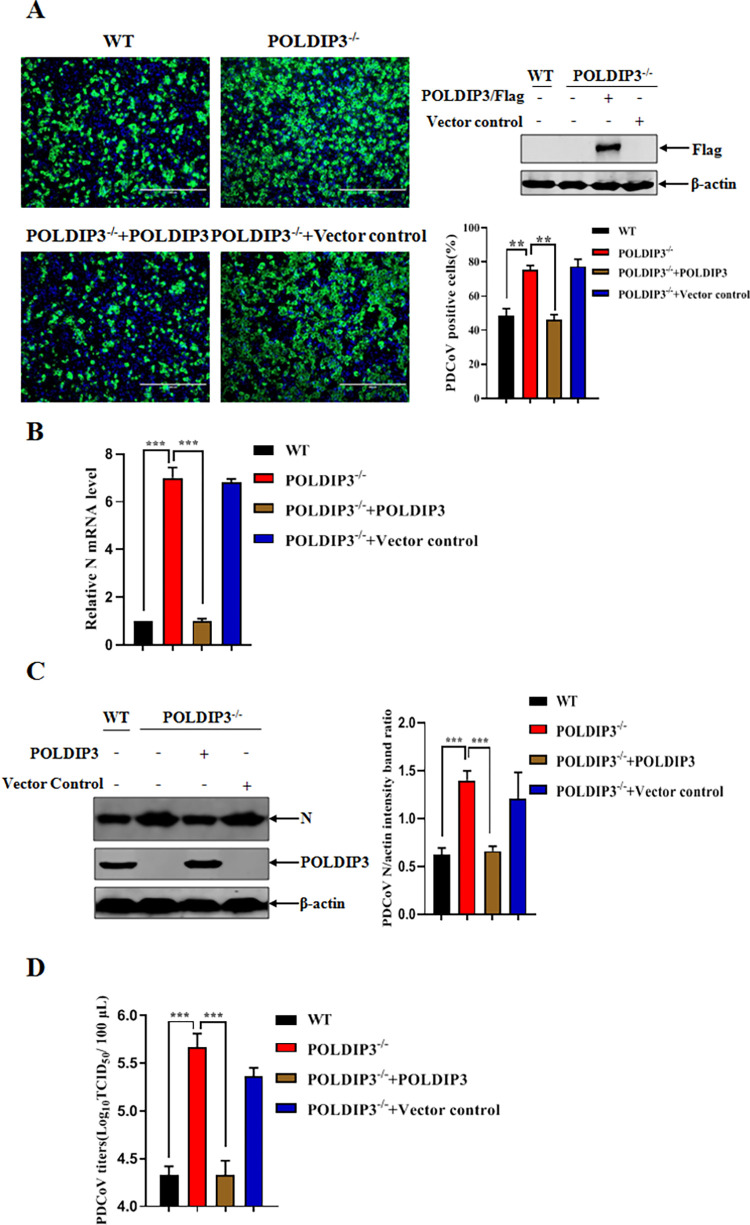
Complementation expression of POLDIP3 disrupts PDCoV replication in POLDIP3^-/-^ cells. (A) POLDIP3 complementation inhibits PDCoV replication in POLDIP3^-/-^ cells by IFA. POLDIP3^-/-^ cells were transduced with POLDIP3 or the vector control described above and then infected with PDCoV at an MOI of 1 for 24 h. Meanwhile, another WT and POLDIP3^-/-^ cells were left as controls with only PDCoV infection at an MOI of 1 for 24 h. The percentage of PDCoV-infected cells was determined by IFA, followed by the confirmation of POLDIP3 expression driven by lentiviruses in POLDIP3^-/-^ cells by Western blotting. (B) Complementation of POLDIP3 suppresses PDCoV replication in POLDIP3^-/-^ cells by RT-qPCR. The target cells were treated as previously mentioned in (A), and the mRNA levels of the PDCoV N gene were determined by RT-qPCR. (C and D) Ectopic expression of POLDIP3 results in the inhibition of PDCoV infection in POLDIP3^-/-^ cells. The target cells were treated as previously mentioned in (A), followed by comparisons of PDCoV replication by Western blotting (C) and TCID_50_ (D)_,_ respectively. The results represent three independent experiments (the means ± SD). *, *P*<0.05, **, *P*<0.01, ***, *P*<0.001. The *P* value was calculated using Student’s t-tests.

To corroborate our previous findings, POLDIP3 expression was restored in POLDIP3^-/-^ cells using the lentiviruses expressing porcine POLDIP3. The POLDIP3^-/-^ cells were transduced with lentiviruses expressing the POLDIP3 gene or vector control for 24 h, the cells were inoculated with PDCoV and cultured for an additional 24 h. PDCoV infection was substantially inhibited in POLDIP3^-/-^ cells with lentivirus-mediated complementation expression of POLDIP3 compared to POLDIP3^-/-^ cells with lentivirus control, confirming the antiviral activity of POLDIP3 against PDCoV infection (Fig 5A). An obvious decrease in PDCoV N mRNA amount was proven by RT-qPCR analysis in POLDIP3^-/-^ cells with overexpression of POLDIP3 relative to POLDIP3^-/-^ cells with mock treatment (Fig 5B). Furthermore, PDCoV replication was inhibited followed by ectopic expression of POLDIP3 in POLDIP3^-/-^ cells, as detected by Western blotting to evaluate the levels of viral N protein (Fig 5C). Consistent with the above results, an apparent decrease in progeny virus was further determined by TCID_50_ assay in the PDCoV-infected POLDIP3^-/-^ cells with POLDIP3 complementation (Fig 5D). Taken together, POLDIP3 complementation restores the inhibitory effects of POLDIP3 in the POLDIP3^-/-^ cells, confirming POLDIP3 as an innate antiviral regulator against PDCoV infection.

### PDCoV nsp5 cleaves POLDIP3 via 3C-like protease activity

**Fig 6:**
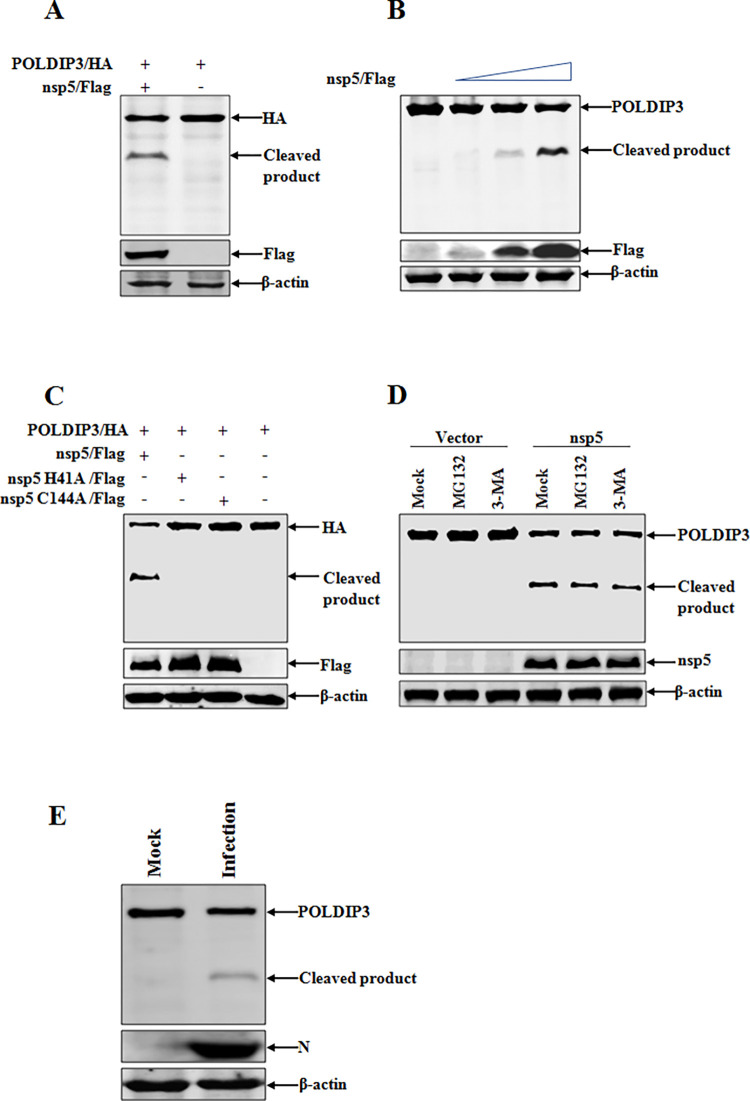
PDCoV nsp5 cleaves POLDIP3 through its protease activity. (A) PDCoV nsp5 cleaves POLDIP3. IPEC-J2 cells were co-transfected with plasmids of POLDIP3-HA and PDCoV nsp5-Flag or empty vector, and then cell samples were collected at 30 h post-transfection for Western blotting analysis. (B) POLDIP3 cleavage in a PDCoV nsp5 dose-dependent manner. IPEC-J2 cells were co-transfected with POLDIP3-HA and increased quantities of PDCoV nsp5-Flag or empty vector. Cell lysates were prepared and analyzed by Western blotting at 30 h post-transfection. (C) PDCoV nsp5 cleaves POLDIP3 depending on its protease activity. IPEC-J2 cells were transfected with POLDIP3-HA along with the wild-type PDCoV nsp5-Flag (nsp5) or its protease-defective mutant (nsp5 H41A or nsp5 C144A). After 30 h post-transfection, cells were collected and lysed for Western blotting. (D) PDCoV nsp5-mediated cleavage of POLDIP3 independent of proteasome degradation and autophagy. IPEC-J2 cells transfected with POLDIP3-HA and PDCoV nsp5-Flag were treated with MG132 (10 μM) or 3-MA (5 mM), followed by detection of cleavage by Western blotting. (E) PDCoV infection induces POLDIP3 cleavage. IPEC-J2 cells were infected with PDCoV and harvested to detect POLDIP3 expression and cleavage at 24 h post-infection.

Previous results demonstrated that POLDIP3 was downregulated followed by PDCoV infection and we then questioned the mechanism of PDCoV-mediated reduction of POLDIP3. Objectively, a suspected cleavage band was discovered when IPEC-J2 cells were co-transfected with porcine POLDIP3-HA and PDCoV nsp5-Flag plasmids followed by Western blotting with antibodies against HA or Flag tag (Fig 6A), suggesting that PDCoV encoded nsp5 is involved in the cleavage of POLDIP3 to reduce its expression as previously reported activities of 3C-like protease (3CLpro) [32]. To further confirm the possible cleavage mediated by PDCoV nsp5, POLDIP3-HA was co-transfected with increasing PDCoV nsp5 into IPEC-J2 cells. As shown in Fig 6B, PDCoV nsp5 could cleavage POLDIP3 dose-dependently, leaving a cleaved product (~30 kDa) from the C-terminal of POLDIP3.

As the nsp5 of CoV contains the catalytic residues His41 and Cys144 (numbering based on PDCoV nsp5), any mutation could disrupt protease activity [33, 34]. To characterize the critical roles of His41 and Cys144 of PDCoV nsp5 in cleavage of POLDIP3, cells were co-transfected with POLDIP3-HA and WT nsp5 or mutation (nsp5 H41A or nsp5 C144A) plasmid of PDCoV nsp5 fused with a Flag tag at the C-terminal. In contrast to the activity of WT nsp5, the POLDIP3 cleavage mediated by nsp5 was completely abolished upon the transfection of nsp5 H41A or nsp5 C144A (Fig 6C), implying that PDCoV nsp5 cleaves POLDIP3 in a 3CLpro dependent manner. Meanwhile, we sought to investigate the involvement of proteasome degradation and autophagy in the PDCoV nsp5-mediated downregulation of POLDIP3. Cells were co-transfected with POLDIP3-HA and nsp5 or vector control followed by treatment with the proteasome inhibitor MG132 or the autophagy inhibitor 3-MA. PDCoV nsp5 mediated POLDIP3 cleavage was not affected by these inhibitors, indicating the independence of proteasome and cellular autophagy pathways in POLDIP3 cleavage by PDCoV nsp5 (Fig 6D). Consistent with the cleavage of POLDIP3 by PDCoV nsp5, reduction in endogenous POLDIP3 protein was observed, and an expected cleavage product was also detected in PDCoV infected IPEC-J2 cells, confirming the biological effect of this cleavage during PDCoV infection (Fig 6E).

### PDCoV nsp5 cleaves POLDIP3 at residue Q176

**Fig 7:**
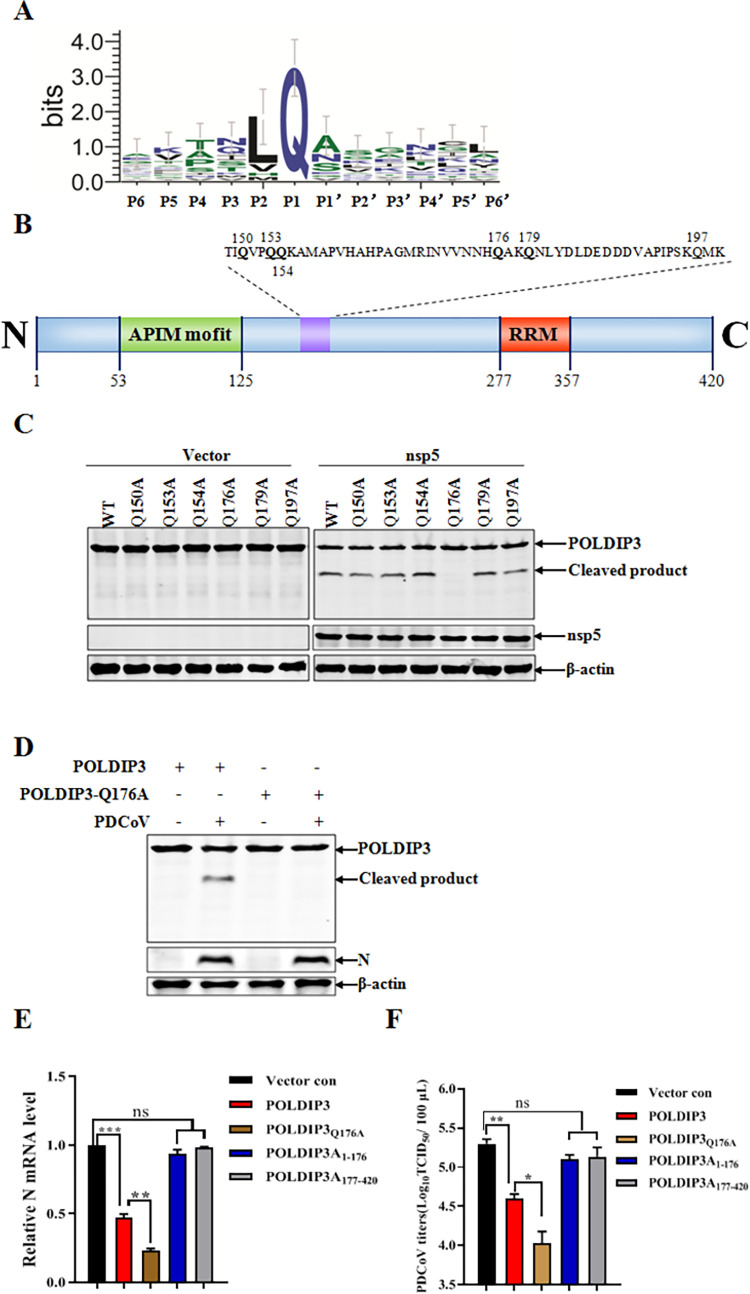
PDCoV nsp5 cleaves POLDIP3 at residue glutamine 176 (Q176). (A) Logo analysis of the cleavage site predicted by WebLogo (http://weblogo.threeplusone.com/), based on the polyprotein and other substrates cleavage of PDCoV nsp5. (B) Schematic representation of POLDIP3 and its mutant sites. (C) Identification of the recognition site in PDCoV nsp5-mediated cleavage of POLDIP3. IPEC-J2 cells were transfected with nsp5-Flag and POLDIP3-HA or POLDIP3 mutants, including POLDIP3_Q150A_, POLDIP3_Q153A_, POLDIP3_Q154A_, POLDIP3_Q176A_, POLDIP3_Q179A_ and POLDIP3_Q197A_. At 30 h post-transfection, cell lysates were prepared and analyzed by Western blotting. (D) Q176A is required for POLDIP3 cleavage in the context of PDCoV infection. Wild-type POLDIP3-HA or POLDIP3-HA_Q176A_ mutant was transfected into IPEC-J2 cells for 24 h, followed by PDCoV infection for another 24 h to analyze the POLDIP3 cleavage by Western blotting. (E and F) POLDIP3 cleavage abolishes its antiviral activity. IPEC-J2 cells were transfected with plasmids encoding wild-type POLDIP3, POLDIP3_Q176A_, POLDIP3_1–176_, POLDIP3_177–420_ or empty vector for 24 h and then infected with PDCoV for another 24 h. The PDCoV N mRNA and viral titer were determined by RT-qPCR (E) or TCID50 assay (F). The results represent three independent experiments (the means ± SD). *, *P*<0.05, **, *P*<0.01, ***, *P*<0.001. The *P* value was calculated using Student’s t-tests.

To identify the recognition site of POLDIP3 in PDCoV nsp5-mediated cleavage, the substrate specificity of PDCoV nsp5 in target proteins was analyzed (S2 Fig). As shown in Fig 7A, a specific preference for substrate cleavage by CoV nsp5 has been previously documented as glutamine (Gln [Q]) at the P1 position [35, 36]. First, we analyzed the potential cleavage site by using the online NetCorona-1.0 software (https://services.healthtech.dtu.dk/service.php?NetCorona-1.0). The glutamine at the position of 176 (Q176) was predicted to be the potential cleavage site (S3A–S3C Fig). Combined with the molecular weight of the POLDIP3 cleavage product (~30 kDa) by PDCoV nsp5, we speculated that glutamines around Q176 were the potential cleavage sites of POLDIP3 by PDCoV nsp5. As such, a series of POLDIP3 mutants were constructed in which the Gln at each potential P1 position around Q176 was replaced with alanine (Ala [A]) and examined their cleavage by PDCoV nsp5 (Fig 7B). As shown in Fig 7C, PDCoV nsp5-mediated POLDIP3 cleavage was not affected by Q150A, Q153A, Q154A, Q179A, or Q197A mutant. In contrast, the Q176A mutant blocked the appearance of the cleavage product (Fig 7C, lower panel, lane 12), suggesting Q176 was the critical site for POLDIP3 cleavage by PDCoV nsp5. Moreover, the cleavage of POLDIP3 or POLDIP3_Q176A_ was further evaluated in the context of PDCoV infection. Expectedly, the cleaved product of POLDIP3 was also detected in IPEC-J2 cells with ectopic expression of POLDIP3 followed by PDCoV infection, demonstrating that exogenous POLDIP3 was also subject to cleaving by PDCoV infection (Fig 7D).

To investigate whether PDCoV nsp5-induced cleavage affects the antiviral activity of POLDIP3, two truncated mutants POLDIP3_1-176_ and POLDIP3_177-420_, were constructed, respectively. Antiviral activities of wild-type POLDIP3 or its mutants were examined in IPEC-J2 cells. The target cells were transfected with different constructs, including wild-type POLDIP3, POLDIP3_Q176A_, POLDIP3_1-176_, POLDIP3_177-420_, or empty vector for 24 h followed by PDCoV infection for another 24 h. As shown in Fig 7E, no obvious differences in PDCoV N mRNA were observed in cells transfected with POLDIP3_1-176_, POLDIP3_177-422_, and empty vector, indicating that PDCoV nsp5-mediated cleavage abolished the antiviral activities of wild-type POLDIP3. Compared with wild-type POLDIP3, POLDIP3_Q176A_, which resists PDCoV nsp5-mediated cleavage, exhibited a stronger antiviral effect. These observations were further confirmed by TCID_50_ assay (Fig 7F). Overall, Q176 was the critical site for PDCoV nsp5-mediated POLDIP3 cleavage, which impaired the antiviral activity of POLDIP3 against PDCoV infection.

### PDCoV infection induces endogenous POLDIP3 reduction *in vivo*

**Fig 8:**
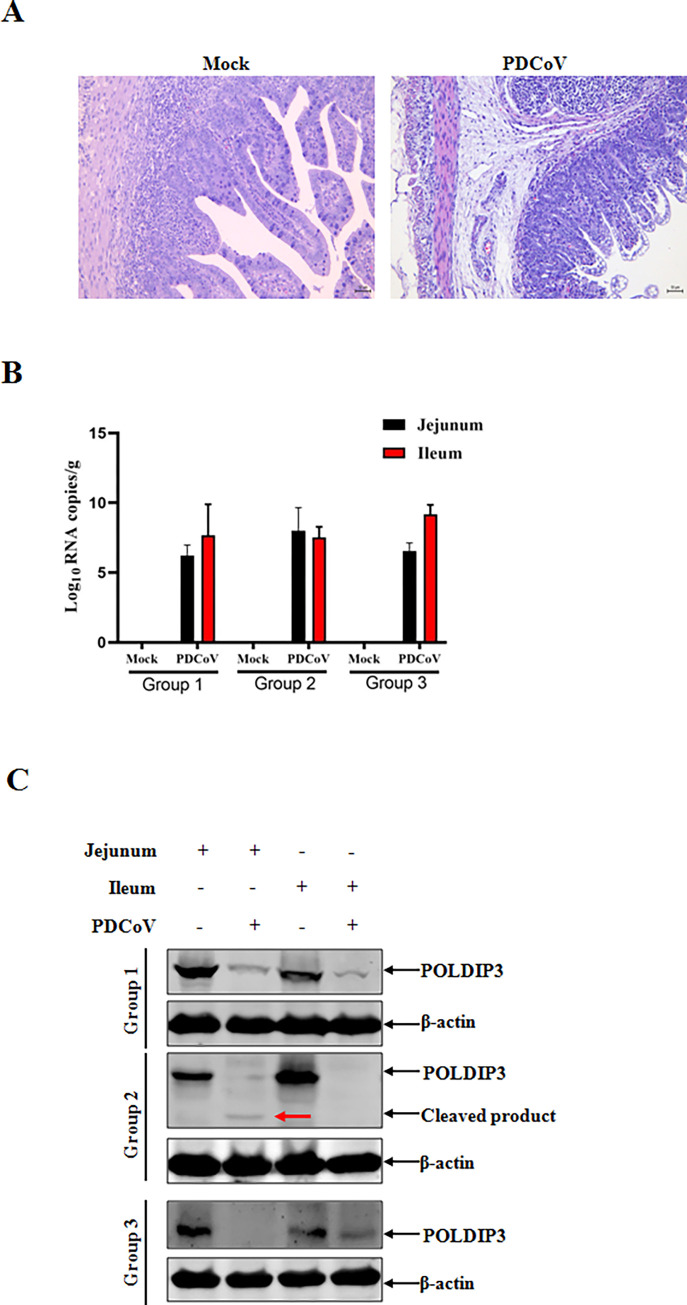
PDCoV infection results in a reduction of endogenous POLDIP3 *in vivo*. (A) histological lesions by H&E staining. Original magnification, ×400. (B) Identification of PDCoV infection. Representative samples of jejunum and ileum were collected and subjected to the detection of PDCoV N mRNA by RT-qPCR. (C) The reduction of endogenous POLDIP3 was confirmed *in vivo* by Western blotting. The endogenous POLDIP3 expressions were evaluated in jejunum and ileum from PDCoV- and mock-infected SPF piglets by Western blot analysis. The cleavage product was indicated by the red arrow.

The accumulated evidence demonstrated that PDCoV infection could antagonize host antiviral response by nsp5-mediated cleavage of endogenous POLDIP3 *in vitro*. However, whether PDCoV infection can induce reductions in POLDIP3 expression *in vivo* remains unclear. Within 2 days post-inoculation (DPI), all piglets orally fed with PDCoV experienced varying degrees of diarrhea and vomiting. At 2 DPI, all the piglets were euthanized for pathological examination, and the different intestinal tissues (jejunum, ileum) were collected for histopathological examination by hematoxylin and eosin (H&E) staining, detection of viral RNA by RT-qPCR and determination of endogenous POLDIP3 expression by Western blotting. The thin-walled intestinal structure was observed with water-like content. Conversely, no obvious clinical signs were present in the mock-infected pigs throughout the experiment. The H&E results indicated that severe atrophic enteritis was clearly observed in the jejunum of PDCoV-infected piglets compared with the mock-infected piglets (Fig 8A). Consistently, high copies of PDCoV N mRNA were identified by detection of RT-qPCR (Fig 8B). Compared with mock-infected piglets, POLDIP3 reduction with the cleaved band was also confirmed in intestinal tissues of PDCoV infected piglets by Western blotting (Fig 8C), indicating that PDCoV infection can aslo induce POLDIP3 downregulation by cleavage *in vivo*.

### POLDIP3 is a common substrate of different coronaviruses nsp5 to antagonize its antiviral activity

**Fig 9:**
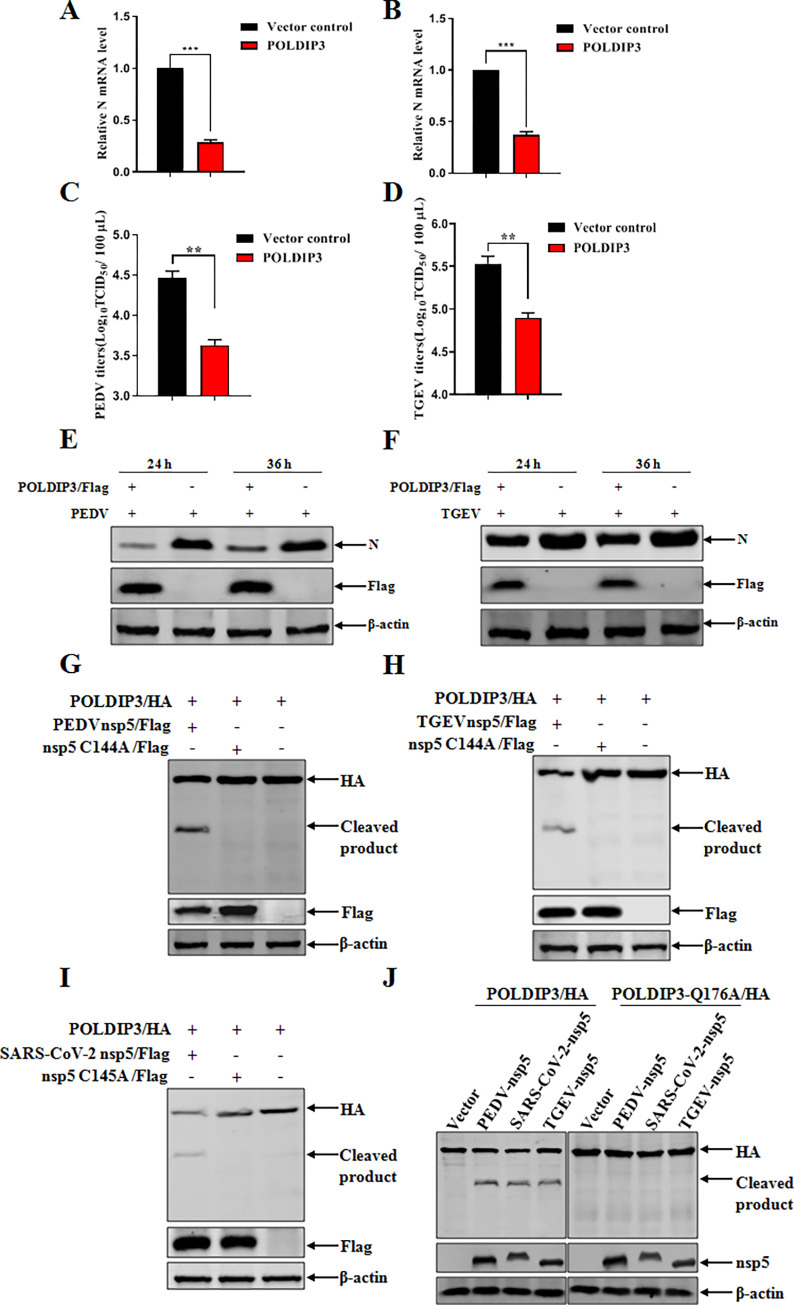
POLDIP3 is a common substrate of different coronaviruses nsp5. (A and B) Ectopic expression of POLDIP3 inhibited PEDV and TGEV replication by RT-qPCR. POLDIP3 proteins were overexpressed for 24 h, and then cells were inoculated with PEDV or TGEV. The levels of PEDV (A) and TGEV (B) N mRNA were determined at 24 h post infection by RT-qPCR. (C and D) Overexpression of POLDIP3 decreased PEDV and TGEV infection by TCID_50_ assay. Cells were subjected to PEDV (C) and TGEV (D) infection for 24 h following POLDIP3 overexpression for 24 h. The virus yield was measured by TCID_50_. (E and F) PEDV and TGEV N proteins were inhibited by POLDIP3 overexpression by Western blotting analysis. The target cells were treated as previously mentioned in (A), and PEDV (E) and TGEV (F) replications were evaluated by detecting N proteins by Western blotting. (G to I) Nsp5s from PEDV, TGEV and SARS-CoV-2 harbor the conserved function to cleavage porcine and human POLDIP3. The target cells were transfected with specific POLDIP3-HA along with plasmids encoding Flag-tagged nsp5s or their catalytic defective mutants from PEDV (C144A) (G), TGEV (C144A) (H) and SARS-CoV-2 (C145A) (I), respectively. At 24 h post-transfection, cells were lysed and detected by immunoblotting. (J) Nsp5s from PEDV, TGEV and SARS-CoV-2 cleavage POLDIP3 at the same conserved site. The target cells were co-transfected with wild-type POLDIP3-HA or mutant (POLDIP3-HA_Q176A_) together with nsp5 plasmids from PEDV, TGEV or SARS-CoV-2. After 24 h, cells were lysed and detected by immunoblotting. The results represent three independent experiments (the means ± SD). *, *P*<0.05, **, *P*<0.01, ***, *P*<0.001. The *P* value was calculated using Student’s t-tests.

Subsequently, we then asked whether POLDIP3 exerted antiviral activity on other coronaviruses associated with the clinical diarrhea of piglets. The replications of PEDV and TGEV were evaluated followed by the overexpression of POLDIP3. The ectopic POLDIP3 resulted in significant decrease in the levels of N mRNA of PEDV and TGEV, respectively (Fig 9A and 9B). Furthermore, the replications of PEDV (Fig 9C) and TGEV (Fig 9D) were also inhibited by the overexpression of POLDIP3 when determined by TCID_50_ assay. Consistent with the mentioned results above, the Western blotting data further confirmed the inhibitory effects of POLDIP3 on the replication of PEDV (Fig 9E) and TGEV (Fig 9F), respectively. As expected, POLDIP3 deletion resulted in apparent increase of replications of PEDV and TGEV, respectively (S4 Fig). Next, we questioned whether nsp5 of other CoVs could cleave POLDIP3 as PDCoV nsp5 did, and whether POLDIP3 cleavage is species-restricted. It has been reported that cysteine (Cys) residues of CoVs nsp5 form a catalytic dyad, and the cysteine mutation can disrupt its protease activity [37]. In addition, previous studies have demonstrated that nsp5 cleavage is not species-dependent and PEDV nsp5 C144A, TGEV nsp5 C144A and SARS-CoV-2 nsp5 C145A do not show protease activity [37, 38]. Multiple-sequence alignment showed that nsp5 of PEDV, TGEV and SARS-CoV-2 are highly similar to PDCoV nsp5 (S5 Fig), especially in their catalytic domains (S6 Fig), and the amino acid sequences of POLDIP3 from different mammalian species were highly conserved (S7 Fig), particularly, the Q176 residue recognized by PDCoV nsp5 is identical among POLDIP3 of different mammalian species (S8 Fig). Thus, the wild-type or the protease-defective nsp5 of PEDV, TGEV and SARS-CoV-2 was co-transfected with the corresponding POLDIP3, and the results showed that all these wild-type nsp5 proteins cleaved POLDIP3 but not the protease-defective nsp5 (Fig 9G–9I). Likewise, similar results were observed for cleavage of the corresponding POLDIP3 by PEDV, TGEV and SARS-CoV-2 nsp5 at Q176 (Fig 9J). The results described above suggest that POLDIP3 exerted broad antiviral activities for different coronaviruses (e.g., PDCoV, PEDV and TGEV) and the nsp5 from PDCoV, PEDV, TGEV and SARS-CoV-2 can cleave the corresponding POLDIP3 at the same Q176 site. Collectively, a pan-antagonism mechanism was identified to evade the host cell antiviral responses among different coronaviruses.

## Discussion

The innate immune system is the first line of the host defense program against pathogens and harmful substances. The activated innate immune system produces IFNs and cytokines that perform antiviral functions to eliminate invading viruses [39–42]. However, during the coevolution with their host, viruses have developed diverse strategies to evade host antiviral defense programs to benefit their replications [43–46]. Therefore, understanding the interaction between virus and the host can not only clarify the infection process of virus in host cells, but also help discover unknown pathogenesis of virus infections.

Coronaviruses have acquired multiple mechanisms to antagonize the host innate immune system by targeting cellular pattern recognition receptors or blocking downstream antiviral signaling molecules. For example, nsp1 proteins of severe acute respiratory syndrome coronavirus (SARS-CoV-1), Middle East respiratory syndrome coronavirus (MERS-CoV), murine hepatitis virus (MHV), TGEV, and PEDV suppress the expressions of host genes [47–49]. The N proteins of PDCoV and swine acute diarrhea syndrome coronavirus (SADS-CoV) mediate K63-linked ubiquitination of porcine RIG-I, thereby inhibiting the host IFN-β production [50, 51]. As well known, an antiviral state was established following the ISGs inductions upon IFNs activation, leading to robust defenses against viral infection. To combat these antiviral effects of ISGs, some proteins encoded by CoVs, such as PEDV N protein, PEDV nsp1, PDCoV nsp5, PDCoV nsp6, and MHV nsp15, have been demonstrated to hijack IFN signaling to reduce ISG production indirectly [32, 52–54]. Of the several known viral evasion strategies, the cleavage of crucial innate immune molecules, including adaptors, kinases, and transcriptional factors, is a common strategy for viruses to escape the innate immune response. The 3C-like protease nsp5 of PEDV, PDCoV, and feline infectious peritonitis virus (FIPV), disrupts type I IFN signaling by cleaving the nuclear transcription factor kappa B essential modulator (NEMO) [34, 55, 56]. PDCoV nsp5 antagonizes type I IFN signaling by cleaving STAT2, an essential factor for IFN-activated JAK-STAT responses [54]. In addition, PDCoV nsp5 cleaves histone deacetylase 2 (HDAC2) to attenuate its antiviral activity [32]. Cleavage of IRF3 and critical modulators of inflammatory pathways (NLRP12 and TAB1) was proven by proteases nsp3 and nsp5 in SARS-CoV-2 infected 293T-ACE2 cells, which was involved in the pathophysiology of COVID-19 [2]. A recent study demonstrated that nsp5 from different coronaviruses, SARS-CoV-2, MERS-CoV, PDCoV, and PEDV, can sustain their infection by suppression pyroptosis via pyroptosis by cleaving gasdermin D (GSDMD) [37]. This study found that PDCoV nsp5 can target POLDIP3 for cleavage in a 3CLpro-dependent manner, independent of proteasome degradation pathway and cell autophagy. Furthermore, subsequent results demonstrated that endogenous POLDIP3 was similarly cleaved in the context of PDCoV infection in the IPEC-J2 cells. Consistent with the POLDIP3 cleavage by nsp5 *in vitro*, reduction in endogenous POLDIP3 by cleavage was further corroborated in intestinal tissues from PDCoV-challenged SPF pigs *in vivo*. Nsp5s from PEDV and TGEV can also cleave porcine POLDIP3. In addition, nsp5 from SARS-CoV-2 can cleave human POLDIP3. Combined with the previous studies, CoV nsp5 has been verified as one of the most convincing and important antagonists against the antiviral signaling pathway of hosts. Based on the robust antagonization against host innate immune response, the CoV nsp5 provides an excellent potential target for innovative strategies and directions to develop effective therapeutics against the coronavirus infections [57]. So far, considerable efforts have been spent characterizing the structure of the protease domains of nsp5, opening a view to the development of medicine or inhibitors [58–60].

POLDIP3 was a novel identified protein interacting with the p50 small subunit of human DNA polymerase δ (polδ). Since the discovery of POLDIP3, relevant studies about POLDIP3 have mainly assessed its role in the occurrence of diverse diseases, including cancer [25]. In contrast to the implications of POLDIP3 in diseases, less information has been reported on the interplay between POLDIP3 and microbial infections. Here, we first identified the involvement of POLDIP3 in PDCoV infection, which was confirmed in the IPEC-J2 cells by iTRAQ and Western blotting analysis. Overexpression of POLDIP3 inhibits PDCoV infection, whereas CRISPR-Cas9 mediated POLDIP3 KO resulted in significant increases in PDCoV replication, implying the antiviral effects of POLDIP3 on PDCoV infection. Collectively, POLDIP3 was involved in positive regulations of host antiviral response.

**Fig 10:**
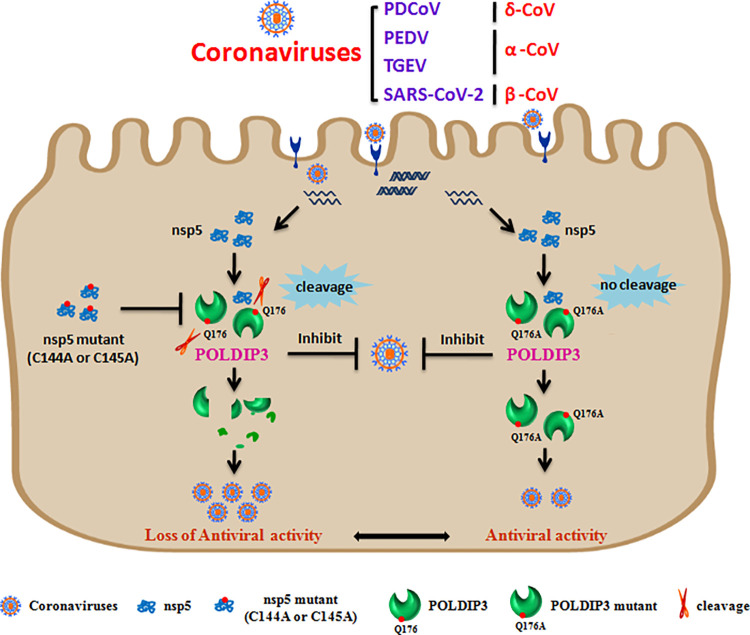
Mechanistic diagram illustrating antagonization of POLDIP3-mediated antiviral activity by coronaviruses nsp5. Coronaviruses nsp5-mediated cleavage abolishes the antiviral activity of POLDIP3 to evade host cell innate immune response.

In summary, we demonstrated that POLDIP3 as a novel antiviral regulator could negatively regulate CoVs infection. Moreover, cleaving POLDIP3 may be a common strategy used by different mammalian CoVs to antagonize the antiviral role of POLDIP3 (Fig 10). Our findings enrich the diversity and similarity of the antagonistic strategies evolved by CoVs infection and facilitate the understanding of CoVs pathogenesis.

## Materials and methods

### Ethics statement

The animal experiment was approved by the Harbin Veterinary Research Institute and performed following animal ethics guidelines and approved protocols (SYXK-2018-032).

### Cell culture and viruses

IPEC-J2 cells (a porcine small intestinal epithelial cell line) and HEK293T cells (human embryonic kidney epithelial cell line) were cultured in Dulbecco’s minimum essential medium (DMEM) (Life Technologies, USA) supplemented with 10% heat-inactivated fetal bovine serum (FBS) (Gibco, USA) at 37°C under 5% CO_2_. PDCoV strain NH (Passage 10, GenBank accession No: KU981062), PEDV strain LNCT (GenBank accession No: KT323980), and TGEV strain AHHF (GenBank accession No: KX499468) were prepared and stored in our laboratory.

### Plasmids and antibodies

The full-length sequences of porcine and human POLDIP3 were amplified from the target cells, respectively, and then cloned into a pCAGGS vector containing a C-terminal hemagglutinin (HA) tag. POLDIP3 substitution mutants, including POLDIP3_Q150A_, POLDIP3_Q153A_, POLDIP3_Q154A_, POLDIP3_Q176A_, POLDIP3_Q179A_, POLDIP3_Q197A_, POLDIP3_1-176_, and POLDIP3_177-420_, were fused with an HA tag in the C-terminal and constructed into pCAGGS vector (Table 1). The constructed nsp5 of CoVs with a C-terminal Flag tag in pCAGGS vector were stocked in our laboratory. The listed antibodies were used in this study, including the POLDIP3 rabbit polyclonal antibody (pAb) from Abcam; Mouse anti-Flag mAb, Mouse anti-HA mAb, and Mouse anti-β-actin mAb were purchased from Sigma; IRDye-conjugated secondary antibody was purchased from Li-Cor Biosciences; The mouse monoclonal antibody against PDCoV N was prepared and preserved in our laboratory.

**Table 1:** Primers used in this study.

Primers	Forward or reverse (5’→3’)	Sequence
**Construction of recombinant plasmids in pCAGGS:**		
POLDIP3^a^ (porcine)	Forward	TTC**ATCGAT**ATGGCGGACATCTCCCTGGACGAGCTCAT
	Reverse	GGA*AGATCT*TCAAGCGTAATCTGGAACATCGTATGGGTAAAGTTTGATTTTGAATTCTGTGG
POLDIP3^a^ (human)	Forward	TTC**ATCGAT**ATGGCGGACATCTCCCTGGACGAACTCAT
	Reverse	GGA*AGATCT*TCAAGCGTAATCTGGAACATCGTATGGGTAAAGCTTGATTTTGAATTCTGTGG
POLDIP3_Q150A_^b^ (porcine)	Forward 1	TTC**ATCGAT**ATGGCGGACATCTCCCTGGACGAGCTCAT
	Reverse 1	CTTCTGCTGTGGAACCGCGATGGTTTTAGTGA
	Forward 2	TCACTAAAACCATCGCGGTTCCACAGCAGAAG
	Reverse 2	GGA*AGATCT*TCAAGCGTAATCTGGAACATCGTATGGGTAAAGTTTGATTTTGAATTCTGTGG
POLDIP3_Q153A_^b^ (porcine)	Forward 1	TTC**ATCGAT**ATGGCGGACATCTCCCTGGACGAGCTCAT
	Reverse 1	TGCCATGGCCTTCTGCGCTGGAACCTGGAT
	Forward 2	ATCCAGGTTCCAGCGCAGAAGGCCATGGCA
	Reverse 2	GGA*AGATCT*TCAAGCGTAATCTGGAACATCGTATGGGTAAAGTTTGATTTTGAATTCTGTGG
POLDIP3_Q154A_^b^ (porcine)	Forward 1	TTC**ATCGAT**ATGGCGGACATCTCCCTGGACGAGCTCAT
	Reverse 1	TGGTGCCATGGCCTTCGCCTGTGGAACCTG
	Forward 2	CAGGTTCCACAGGCGAAGGCCATGGCACCA
	Reverse 2	GGA*AGATCT*TCAAGCGTAATCTGGAACATCGTATGGGTAAAGTTTGATTTTGAATTCTGTGG
POLDIP3_Q176A_^b^ (porcine)	Forward 1	TTC**ATCGAT**ATGGCGGACATCTCCCTGGACGAGCTCAT
	Reverse 1	TAAATTCTGTTTGGCCGCGTGGTTATTGACG
	Forward 2	CGTCAATAACCACGCGGCCAAACAGAATTTA
	Reverse 2	GGA*AGATCT*TCAAGCGTAATCTGGAACATCGTATGGGTAAAGTTTGATTTTGAATTCTGTGG
POLDIP3_Q179A_^b^ (porcine)	Forward 1	TTC**ATCGAT**ATGGCGGACATCTCCCTGGACGAGCTCAT
	Reverse 1	CAGGTCATATAAATTCGCTTTGGCCTGGTGGT
	Forward 2	ACCACCAGGCCAAAGCGAATTTATATGACCTG
	Reverse 2	GGA*AGATCT*TCAAGCGTAATCTGGAACATCGTATGGGTAAAGTTTGATTTTGAATTCTGTGG
POLDIP3_Q197A_^b^ (porcine)	Forward 1	TTC**ATCGAT**ATGGCGGACATCTCCCTGGACGAGCTCAT
	Reverse 1	GGCTACAAATTTCATCGCTTTACTAGGAATGGG
	Forward 2	CCCATTCCTAGTAAAGCGATGAAATTTGTAGCC
	Reverse 2	GGA*AGATCT*TCAAGCGTAATCTGGAACATCGTATGGGTAAAGTTTGATTTTGAATTCTGTGG
POLDIP3_Q176A_^b^ (human)	Forward 1	TTC**ATCGAT**ATGGCGGACATCTCCCTGGACGAACTCAT
	Reverse 1	ATAAATTCTGTTTGGCCGCGTGGTTATTGACAACA
	Forward 2	TGTTGTCAATAACCACGCGGCCAAACAGAATTTAT
	Reverse 2	GGA*AGATCT*TCAAGCGTAATCTGGAACATCGTATGGGTAAAGCTTGATTTTGAATTCTGTGG
POLDIP3_1–176_ (porcine)	Forward	TTC**ATCGAT**ATGGCGGACATCTCCCTGGACGAGCTCAT
	Reverse	GGA*AGATCT*TCAAGCGTAATCTGGAACATCGTATGGGTACTGGTGGTTATTGACGACATTG
POLDIP3_177–420_ (porcine)	Forward	TTC**ATCGAT**ATGGCCAAACAGAATTTATATGACCT
	Reverse	GGA*AGATCT*TCAAGCGTAATCTGGAACATCGTATGGGTAAAGTTTGATTTTGAATTCTGTGG
**Construction of recombinant plasmids in pLVX-IRES-ZsGreen1**^**c**^:		
pLVX-POLDIP3 (porcine)	Forward	AG**ACTAGT**ATGGCGGACATCTCCCTGGACGAGCTCAT
	Reverse	GAGCGGCCGCTCACTTGTCGTCATCGTCTTTGTAGTCAAGTTTGATTTTGAATTCTGTGG
**Real-time PCR (RT-qPCR)**^**d**^:		
qPDCoV-N	Forward	AGCAACCACTCGTGTTACTTG
	Reverse	CAACTCTGAAACCTTGAGCTG
qPOLDIP3	Forward	CAAAACCATCCAGGTTCCAC
	Reverse	TCCGGGACATGTTGGTCAAG
qβ-actin	Forward	CTTCCTGGGCATGGAGTCC
	Reverse	GGCGCGATGATCTTGATCTTC

^a^A HA tag (underlined) was fused at the carboxyl terminus of POLDIP3 by PCR and was cloned into the Cla I (bold) and BglII(italic) sites in the pCAGGS vector.

^b^ A series of POLDIP3s mutants were constructed with a HA tag (underlined) at the carboxyl terminus of POLDIP3 by fusion PCR (two sets of primers: Forward 1 and Reverse 1 & Forward 2 and Reverse 2) and were cloned into the Cla I (bold) and BglII(italic) sites in the pCAGGS vector

^c^A Flag tag (underlined) was fused at the carboxyl terminus of POLDIP3 by PCR and was cloned into the SpeI(bold) and NotI(italic) sites in the pLVX-IRES-ZsGreen1

^d^Used for relative quantitative PCR.

### Virus infection, cell transfection, and drug treatments

Monolayers of IPEC-J2 cells were infected with PDCoV at an MOI of 1 for 1 h at 37°C. The unbound virus was removed, and cells were maintained in a complete medium for different timepoints until samples were harvested. A direct transfection and a bicistronic lentivirus vector pLVX-IRES-ZsGreen1 expression system (Clontech) was introduced to overexpress the target protein. Cells were either transfected with indicated plasmids using X-tremeGENE transfection reagent according to manufacturer’s instruction (Roche, USA), or transduced with recombinant lentivirus as reported previously [61], followed by subsequent assays at the indicated timepoints. For some experiments, different treatments with proteasome inhibitor MG132 (Sigma), autophagy inhibitor 3-methyladenine (3-MA, Sigma), or carrier DMSO (Sigma) were performed in the target cells as detailed in the corresponding *Figure Legends*.

### CRISPR-Cas9-mediated POLDIP3 gene knockout

SgRNA for CRISPR editing was designed using the online software of E-CRISP (http://www.e-crisp.org/E-CRISP/), and the POLDIP3 knockout cell lines were constructed by CRISPR-Cas9 gene editing technology. Oligonucleotides corresponding to the sgRNA were synthesized and cloned into the Cas9-expressing plasmid lentiCRISPRv2 plasmid. The sgRNA sequence was 5′-CTCAGATGCCCGGCTCAAGC-3′. CRISPR-mediated POLDIP3 knockout clones were generated by transfection into IPEC-J2 cells and screened with puromycin (1.5 μg/mL) for five days prior to single-cell seeding into 96-well plates at a dilution of 0.5 cells/well to achieve a pure knockout cell line. Clones were expanded and screened for protein knockout by sequencing and Western blotting analysis. Genomic DNA was extracted using the TIANamp Genomic DNA Kit (TIANGEN, Beijing) and used as a template for amplifying of an approximate 200~300 bp region flanking the PAM site. Target bands were gel-extracted, followed by the Sanger sequencing method. The identification primers used for genomic amplification were POLDIP3-Forward: 5′-TCCAGCAGAGATTTGATGCC-3′ and POLDIP3-Reverse: 5′-CTGGATGGTTTTAGTGAGCTT-3′.

### Immunofluorescence assay (IFA)

IFA was performed as described previously with slight modification [22]. Briefly, WT or POLDIP3^-/-^ cells were infected with PDCoV for 24 h, and the cells were fixed and stained with antibodies against PDCoV N for 1 h [62]. After the removal of unbound antibodies, the cells were stained with fluorescein isothiocyanate (FITC) or Alexa Fluor 596-conjugated goat anti-mouse IgG (Jackson ImmunoResearch) for another 1 h, followed by nuclei staining with DAPI (4,6-diamidino-2-phenylindole; Sigma). After washing the cells, the fluorescence was visualized with an Olympus inverted fluorescence microscope equipped with a camera.

### Western blotting assay

Western blotting analysis was performed as previously described [63]. Treated samples were lysed in RIPA buffer containing protease inhibitor cocktail and phosphatase inhibitors (Roche, Switzerland), separated by SDS-PAGE under reducing conditions, and transferred onto a PVDF membrane (Merck Millipore, USA). After blocking, the membranes were incubated with a primary antibody and then probed with an appropriate IRDye-conjugated secondary antibody (Li-Cor Biosciences, Lincoln, NE). The membranes were scanned using an Odyssey instrument (Li-Cor Biosciences) according to the manufacturer’s instructions.

### Quantitative reverse transcription–PCR (RT-qPCR)

RT-qPCR analysis was carried out as described previously [64]. Total RNA was extracted from cells and subjected to RT-qPCR using specific primers as listed in Table 1. Relative gene quantification was performed by the method of by the cycle threshold (∆∆CT) method [65].

### TCID_50_ assay

Collected virus samples were frozen and thawed three times and clarified by centrifugation at 8,000×g for 10 min prior to titration. TCID_50_ assays were performed according to the method of Reed & Muench as previously described [66]. Briefly, cell monolayers in 96-well tissue culture plates (Corning, USA) were inoculated with 100 μL each virus stock serially diluted 10-fold for 4 days prior to observation of the presence of a cytopathic effect.

### Cell viability assay

Cell viability was assessed using a cell counting kit-8 (CCK-8) (GLPBIO, USA). Assays were performed according to the manufacturer’s instructions. Briefly, the WT and POLDIP3^-/-^ cells were incubated in 96-well plates, and the cell viabilities were measured at 12 h, 24 h, and 36 h. A total of 10 μL of CCK-8 reagents were added to each well of the plates, and the cells were incubated at 37°C for 1 h, then, the absorbance at 450 nm was measured by a microplate reader.

### Animal experiment

Six 5-day-old specific-pathogen-free (SPF) piglets were purchased from the Experimental Animal Breeding Center of Harbin Veterinary Research Institute and were randomly divided into two groups housed in separated containments. After 1 day of acclimation, piglets were orally challenged with 10 ml of PDCoV (NH strain) at a dose of 1.0 × 10^7^ TCID_50_, while the mock-infected piglets were inoculated with 10 ml of maintenance medium. The clinical signs of vomiting and diarrhea were recorded every 12 h after the PDCoV infection. All the piglets were euthanized at 48 h post-infection. Respective intestinal samples were collected, followed by examinations of pathological and histological changes, detection of PDCoV RNA abundance, and evaluation of endogenous POLDIP3 expression.

### Statistical analysis

All statistical analyses were performed using GraphPad Prism (GraphPad Software, Inc.). The data are expressed as the mean ± SD. A *P* value <0.05 was considered significant.

## Supporting information

S1 Fig Infection of higher passage of PDCoV induced POLDIP3 reduction. IPEC-J2 cells were mock-infected or infected with PDCoV with higher passage (passage 120) at an MOI of 1 and then lysed for detection of endogenous POLDIP3 at indicated timepoints by Western blotting analysis.
(TIF)

S2 Fig Substrate specificity of PDCoV nsp5-mediated cleavage. Cleavage sequences of viral and host substrates by PDCoV nsp5 were collected and analyzed. The glutamine (Q) in the P1 position was conserved among the different substrates.
(TIF)

S3 Fig Prediction of PDCoV nsp5-mediated cleavage site for POLDIP3. (A to C) The nsp5-mediated cleavage sites were predicted by using the online NetCorona-1.0 software (https://services.healthtech.dtu.dk/service.php?NetCorona-1.0) and the glutamine at the position of 176 (Q176) was predicted to be the potential cleavage site.
(TIF)

S4 Fig POLDIP3 deletion promoted the replications of PEDV and TGEV. WT and POLDIP3^-/-^ cells were infected with PEDV and TGEV for 24 h, respectively, and then the samples were analyzed for detection of N mRNA by RT-qPCR.
(TIF)

S5 Fig Percentage identity of the nsp5 protease sequences among different coronaviruses. Sequences of the coronavirus nsp5, including PDCoV, PEDV, TGEV and SARS-CoV-2, were aligned and analyzed by DNAMAN software.
(TIF)

S6 Fig Conserved catalytic residues (Cys) are observed among different coronaviruses nsp5. Nsp5s from PDCoV, PEDV, TGEV and SARS-CoV-2, were collected and the conserved catalytic residue Cys144 (in the red box, numbering based on PDCoV nsp5) was analyzed by the DNAMAN software.
(TIF)

S7 Fig Percentage identity between amino acid sequences of porcine and human POLDIP3. POLDIP3 sequences originated from porcine and human were selected and the divergence of amino acids was analyzed by the DNAMAN software.
(TIF)

S8 Fig Conserved cleavage site of Q176 between porcine and human POLDIP3. POLDIP3 sequences from porcine and human were aligned by the DNAMAN software and the Q176 (referred to the asterisk in the red box) was identified as the conserved cleavage site for nsp5-mediated cleavage.
(TIF)
