# Supplementary figures and images for: Broad antagonism of coronaviruses nsp5 to evade the host antiviral responses by cleaving POLDIP3
Source: PLoS Pathog. 2023 Oct 6;19(10):e1011702. doi: 10.1371/journal.ppat.1011702 (PMC10602385; doi:10.1371/journal.ppat.1011702)

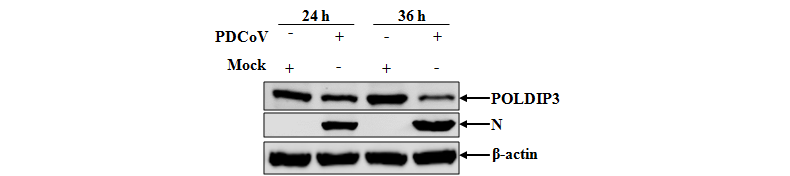

Supplement: S1 Fig — IPEC-J2 cells were mock-infected or infected with PDCoV with higher passage (passage 120) at an MOI of 1 and then lysed for detection of endogenous POLDIP3 at indicated timepoints by Western blotting analysis. (TIF) [file ppat.1011702.s001.tif]

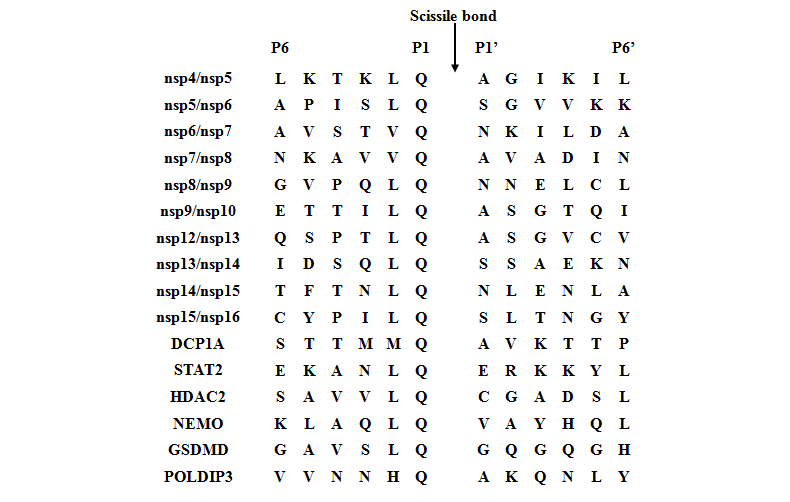

Supplement: S2 Fig — Cleavage sequences of viral and host substrates by PDCoV nsp5 were collected and analyzed. The glutamine (Q) in the P1 position was conserved among the different substrates. (TIF) [file ppat.1011702.s002.tif]

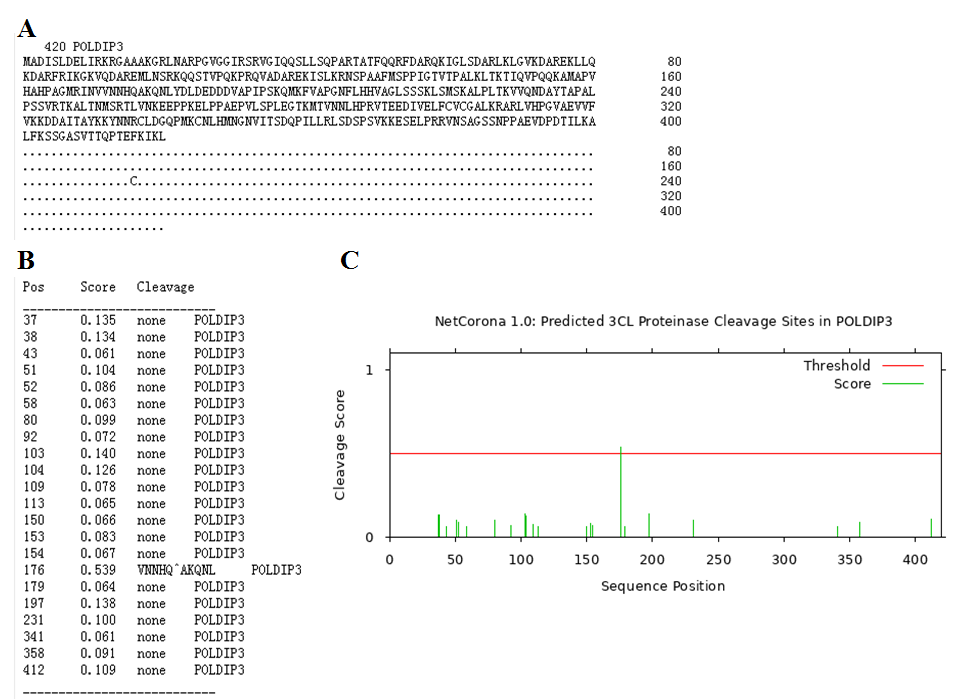

Supplement: S3 Fig — (A to C) The nsp5-mediated cleavage sites were predicted by using the online NetCorona-1.0 software (https://services.healthtech.dtu.dk/service.php?NetCorona-1.0) and the glutamine at the position of 176 (Q176) was predicted to be the potential cleavage site. (TIF) [file ppat.1011702.s003.tif]

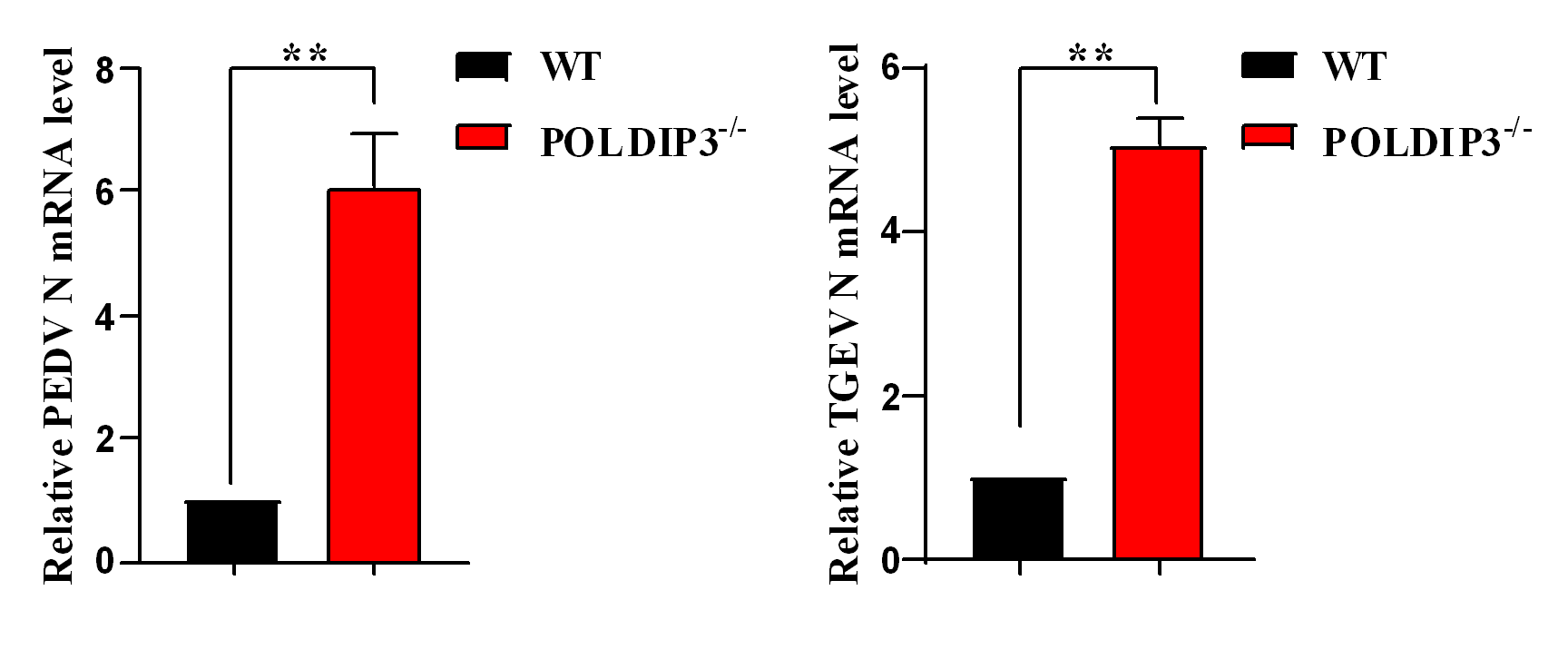

Supplement: S4 Fig — WT and POLDIP3-/- cells were infected with PEDV and TGEV for 24 h, respectively, and then the samples were analyzed for detection of N mRNA by RT-qPCR. (TIF) [file ppat.1011702.s004.tif]

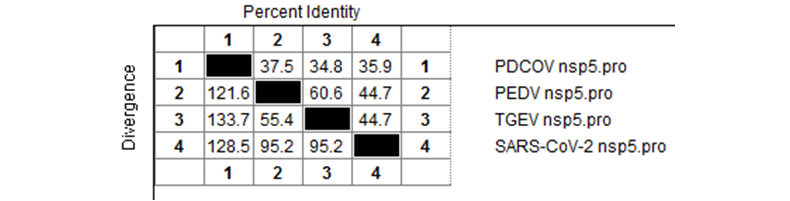

Supplement: S5 Fig — Sequences of the coronavirus nsp5, including PDCoV, PEDV, TGEV and SARS-CoV-2, were aligned and analyzed by DNAMAN software. (TIF) [file ppat.1011702.s005.tif]

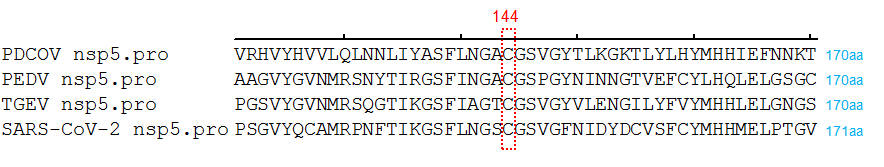

Supplement: S6 Fig — Nsp5s from PDCoV, PEDV, TGEV and SARS-CoV-2, were collected and the conserved catalytic residue Cys144 (in the red box, numbering based on PDCoV nsp5) was analyzed by the DNAMAN software. (TIF) [file ppat.1011702.s006.tif]

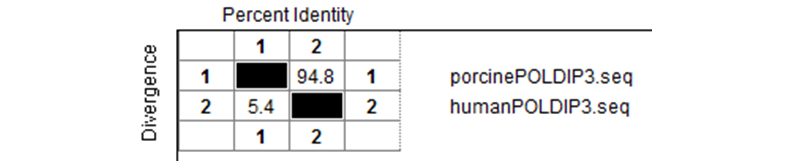

Supplement: S7 Fig — POLDIP3 sequences originated from porcine and human were selected and the divergence of amino acids was analyzed by the DNAMAN software. (TIF) [file ppat.1011702.s007.tif]

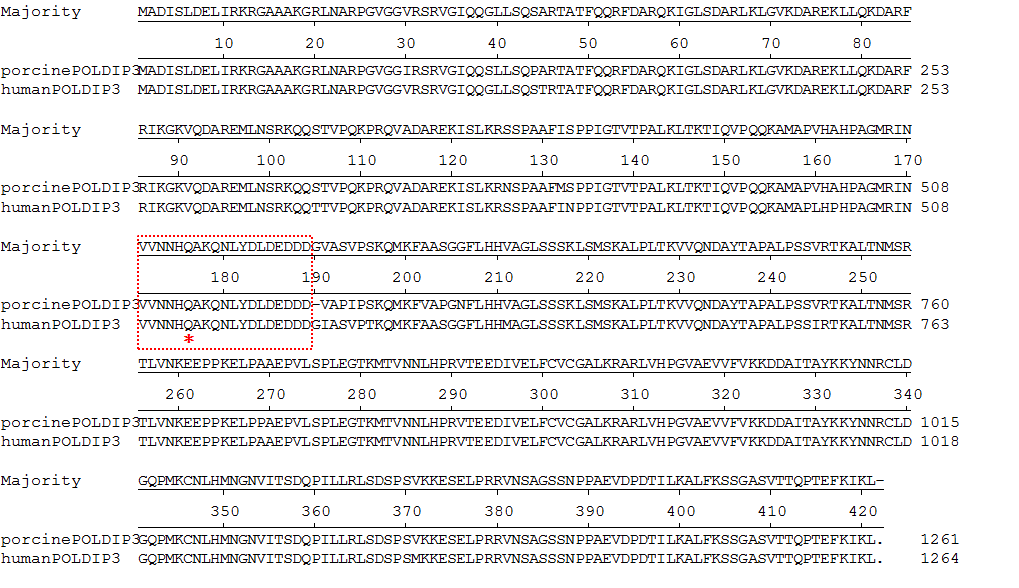

Supplement: S8 Fig — POLDIP3 sequences from porcine and human were aligned by the DNAMAN software and the Q176 (referred to the asterisk in the red box) was identified as the conserved cleavage site for nsp5-mediated cleavage. (TIF) [file ppat.1011702.s008.tif]
